# A Decision Support System for Diabetes Chronic Care Models Based on General Practitioner Engagement and EHR Data Sharing

**DOI:** 10.1109/JTEHM.2020.3031107

**Published:** 2020-10-14

**Authors:** Emanuele Frontoni, Luca Romeo, Michele Bernardini, Sara Moccia, Lucia Migliorelli, Marina Paolanti, Alessandro Ferri, Paolo Misericordia, Adriano Mancini, Primo Zingaretti

**Affiliations:** 1 Department of Information EngineeringUniversità Politecnica delle Marche 9294 60131AnconaItaly; 2 Federazione Italiana Medici di Medicina Generale (FIMMG)00144RomeItaly

**Keywords:** Cloud computing, decision support system, electronic health records, general practice, type 2 diabetes

## Abstract

Objective Decision support systems (DSS) have been developed and promoted for their potential to improve quality of health care. However, there is a lack of common clinical strategy and a poor management of clinical resources and erroneous implementation of preventive medicine. Methods To overcome this problem, this work proposed an integrated system that relies on the creation and sharing of a database extracted from GPs’ Electronic Health Records (EHRs) within the Netmedica Italian (NMI) cloud infrastructure. Although the proposed system is a pilot application specifically tailored for improving the chronic Type 2 Diabetes (T2D) care it could be easily targeted to effectively manage different chronic-diseases. The proposed DSS is based on EHR structure used by GPs in their daily activities following the most updated guidelines in data protection and sharing. The DSS is equipped with a Machine Learning (ML) method for analyzing the shared EHRs and thus tackling the high variability of EHRs. A novel set of T2D care-quality indicators are used specifically to determine the economic incentives and the T2D features are presented as predictors of the proposed ML approach. Results The EHRs from 41237 T2D patients were analyzed. No additional data collection, with respect to the standard clinical practice, was required. The DSS exhibited competitive performance (up to an overall accuracy of 98%±2% and macro-recall of 96%±1%) for classifying chronic care quality across the different follow-up phases. The chronic care quality model brought to a significant increase (up to 12%) of the T2D patients without complications. For GPs who agreed to use the proposed system, there was an economic incentive. A further bonus was assigned when performance targets are achieved. Conclusions The quality care evaluation in a clinical use-case scenario demonstrated how the empowerment of the GPs through the use of the platform (integrating the proposed DSS), along with the economic incentives, may speed up the improvement of care.

## I. Introduction

Type 2 Diabetes (T2D) results from an ineffective use of insulin. The risk of developing T2D depends on an interplay of genetic and metabolic factors. For instance, high waist and body mass index (BMI) are associated with an increased risk, though the relationship may vary in different populations [1]. In 2015, the World Health Organization (WHO) estimated a global prevalence of diabetes around the 9%, with more than 90% of the patients being affected by T2D [2], [3]. Only in 2012, diabetes caused 1.5 million deaths, with more than 8 out of 10 deaths occurring in low and middle income countries. In developing countries, more than half of all diabetes cases goes undiagnosed due to the poor T2D symptoms, at least at the early T2D stage. The WHO anticipates that worldwide deaths from diabetes will double by 2030 [4]. As reported by the International Diabetes Federation (IDF), T2D early diagnosis and treatment can save lives and prevent, or significantly delay, complications [5].

T2D strongly impacts on the costs of national health systems (NHSs). According to the International Diabetes Federation [6], health expenditure for diabetes was estimated at US$105.5 billion in the European Region in 2010 (the 10% of the total health expenditure). This expenditure is expected to reach US$ 124.6 billion by 2030. The estimated costs for the European countries are around the 9%. In Italy, the total cost is about 15 billion euro, with an increasing trend up to the 14.4% in 2040, slightly lower than the one expected at European level (18%) [3], [7]. T2D also causes a significant loss of productivity (work days lost, lower working efficiency, early retirement) and mortality and such social costs represent a heavy economic burden, not always easy to quantify, on society [8].

In Italy many legislative initiatives have taken place on the protection of the diabetic patients, which have merged over time into a National Diabetic Disease Plan (NDDP). Following legal considerations on T2D patient treatment that date back to 1987 [9], the National Diabetic Disease Plan (NDPP) from the Italian Ministry of Health has been released [10]. The NDPP has identified different areas of intervention to standardize treatments of prevention, diagnosis and monitoring of people with T2D living in Italy. The NDPP foresees a capillary network of GPs and other healthcare professionals (nurse, nutritionists, psychologist, podiatrist, cardiologist, nephrologist, neurologist, ophthalmologist, etc.) and provides regular consultation to approximately 50% of people suffering frm T2D. Consequently, one of the major challenges of modern care is to develop and sustain a person-centered management of T2D that relies on interdisciplinary work, communication, data collection, continuous monitoring, and processing and well as reduction of costs.

However, several Italian Regions are independently designing their own models for chronic management and reorganization of territoriality care, with inevitable inhomogeneities. Further barriers to optimal care include limited appointment times, lack of easy access to patient information, and fragmentation of data between healthcare providers. Optimizing the use of the Electronic Health Records (EHRs) by configuring a clinical Decision Support System (DSS), changing workflow patterns to include team management, and implementing a structured patient education can improve the management of T2D in primary care and in successive levels [11], [12].

A wider adoption of EHRs would reduce health care costs, medical errors [13], [14], healthcare disparities, patient complications in hospitals and mortality [15]–[17]. Moreover, sharing EHRs among healthcare professionals will decrease the use of unnecessary services, such as repeated laboratory tests every time the patient changes hospital and office visits [13], [18]. In this scenario, in order to foster the digitalization and sharing of health data in an easy and accessible way, as well as to coordinate data flows, the Netmedica Italia (NMI) has been established, in cooperation between FIMMG (the largest Italian federation of GPs) and Federsanità ANCI (the Italian federation of Public Health Agencies). This was done with the final goal to offer Health Information Technolog (HIT) services to GPs at national level.

Ermakova *et al.* [19] surveyed the use of cloud computing technology in healthcare. The survey pointed out the importance, for GPs, to share healthcare data under a common standard system [20]–[22]. On this consideration, and considering the central role of GPs in effective chronic-disease diagnosis and management strategies, we developed a platform for GPs data sharing and unified T2D patient management, guaranteeing the interoperability (e.g., using EHRs data standards) of the platform with other healthcare databases.

### A. Paper Contributions

This work overcomes solutions in the literature (Sec. II), by developing a novel framework with relevant contributions:

• The proposed solution is based on the standard EHR structure used by GPs in their daily activities, ensuring large-scale use;
• A novel set of quality indicators for shared data and T2D care process quality is presented;
• The framework is equipped with a Machine Learning (ML)- based DSS, analyzing the shared EHRs for T2D screening.
• The architecture involves quality-care evaluation by a second ML approach, with manual annotation on five quality classes;
• The DSS testing was performed on 41237 T2D patients, one order of magnitude larger than the dataset presented in the closer work to ours [23], with real data collected from about 800 GPs;
• A quality-based economic incentive model is proposed to foster GPs empowerment. Up to our knowledge, this is one of the first real applications of quality measures to a standard chronic care model.

The proposed framework is currently used, in the NMI cloud with a Software as a Service (SaaS) design that ensure scalability and real-time performances, with a direct access from GPs ambulatory software in Italy. All Web Services Description Languages (WSDLs) of the proposed EHRs data standard used in this paper are publicly available^1^ and can be considered one of the most comprehensive data standard for GP’s EHR in Europe.^1^http://cloud.fimmg.org/wsdl.php?wsdl

## II. Related Work

### A. EHR Use and Sharing

The adoption of EHRs can represent a possible solution to integrate data provided by different information sources transforming them into useful shared knowledge. This allows to define metrics and assessment of clinical performance as well as to take corrective actions to support better decision-making based on a set of clinical indicators defined to manage the intervention of patients with diabetes.

In a feasibility study within an Italian regional environment, Pecoraro *et al.* [12] show the applicability of a shared EHR in a clinical governance framework. The use of EHRs has the advantage of managing standardized data already integrated in several health infrastructures. An Austrian study [11] underlines as the continuity of care in chronic diseases has a positive impact during the patient follow-up. Yamaguchi and Ito [24] investigated the effectiveness of cooperation of medical experts using data from EHRs in a medium-sized local hospital. Sharing information and electronic clinical path are the main factors in promoting inter-professional work. In addition, introducing an electronic information technology tool, the authors have reported a challenging strategy to improve T2D integrated management. T2D is a chronic and transversal disease related to many other pathologies. Therefore, the integrated management should possess a wide scalability and must be able to discriminate and evaluate other chronic complications that result from T2D [25]. In another work [26], an EHR architecture is used to discriminate prevalence and incidence of cardiovascular disease (CVD) in T2D patients. The large availability of EHR data allows to extract information relevant to favourable or unfavourable long-term strategies related to specific glucose-lowering therapies.

### B. EHR Analysis for DSS

EHR-clinical DSS based have great potential to improve the diabetes care. A systematic review presents the potential clinical, social and economic benefits that a DSS could add to an already existing healthcare system [27], [28]. Clinical guidelines for optimal management of diabetes are widely available, yet adherence to these guidelines remains variable [29]. Clinical DSS systems is designed to guide optimal medical therapy based on individual patient characteristics extracted from the EHR [27]. CDS tools have been developed to provide reminders for routine laboratory testing, recommendations for specific medication choices, and alerts for potential drug-drug interactions. Electronic clinical reminders have evidenced an increased adherence to recommended pharmacotherapy and screening [30]. Holbrook *et al.* [31] showed that when the decision support is shared between physician and patient through a web-based interface, significant improvements in clinical diabetes care can be achieved. In a research conducted by Ati and Omar *et al.* [32], a knowledge based system is created and then integrated with a EHRs database as part of the national E-Health infrastructure. This is used to create a system based on Service Oriented Architecture that is able to predict or monitor the condition of any diabetic patient based on a certain number of features defined by the health authorities.

A widely adopted approach for identifying subjects with and without T2D is to involve experienced physicians that manually design algorithms based on their experience and examination of EHR data [33]–[36]. However, such strategies increasingly prove to be limited and not scalable [33], [34], [36] due to the laborious process of human intervention and rule abstraction capabilities of experts. Furthermore, expert algorithms are often designed with conservative identification strategy, thus may fail to identify complex (e.g., borderline) subjects and miss a significant number of potential T2D cases [37]. Thus, recent work in addition to EHRs has introduced a clinical DSS integrated with a machine learning based framework [38], [39].

Machine learning and data mining models are increasingly utilized in diabetes related research from EHR data. These studies have primarily focused on mining T2D-related EHR data for clinical purposes. For instance, some studies aimed at forecasting clinical risk of diabetes from EHR [40], [41]. Wang *et al.* explain as the use of a shared decision-making (SDM) process in antihyperglycemic medication strategy decisions is necessary due to the complexity of the conditions of diabetes patients. Knowledge of guidelines is used as decision aids in clinical situations, and during this process no patient health conditions are considered. It is proposed a SDM system framework for T2D patients that not only contains knowledge abstracted from guidelines but also employs a multilabel classification model that uses class-imbalanced EHR data and that aims to provide a quality care model to help physicians and patients having a SDM conversation [23], [42] and to improve chronic care models.

## III. A Framework for Decision Process of T2D

**FIGURE 1.**
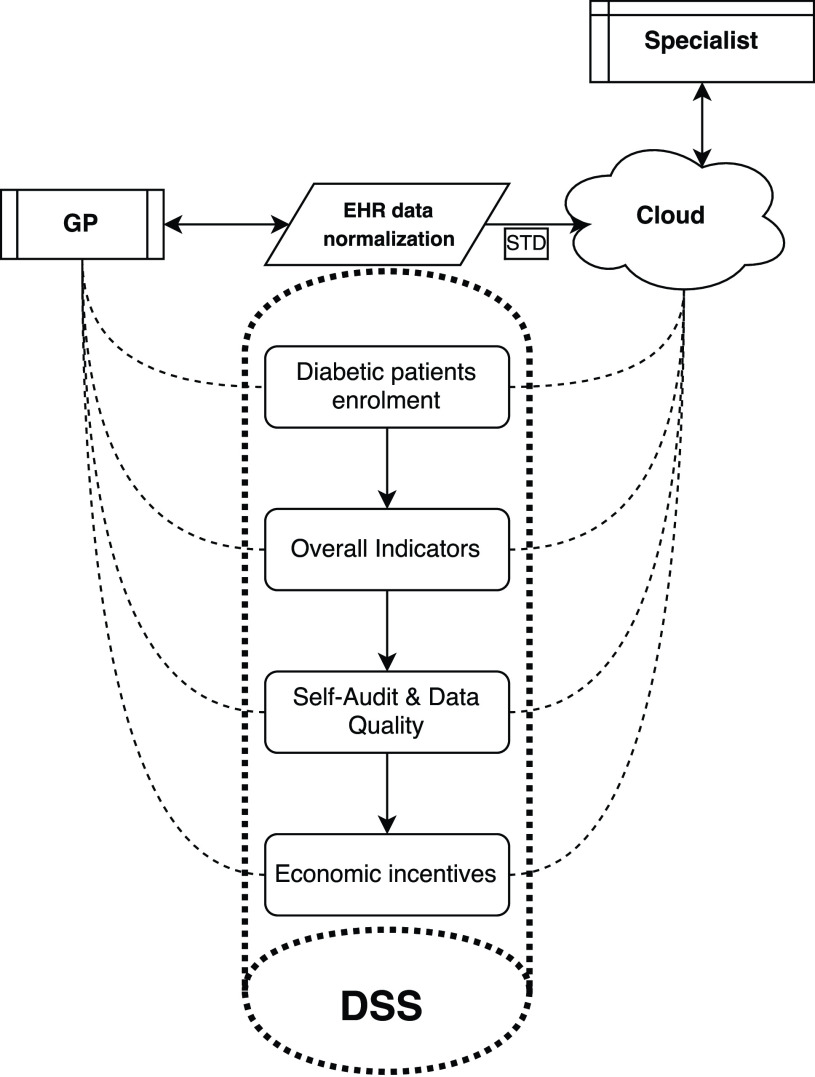
GP’s workflow in T2D integrated management care.

In this section, a DSS framework for T2D is introduced as well as the dataset used for evaluation. The framework is depicted in Figure 1 and comprises five main components:

• Clinical data collection of T2D patients from EHRs and data sharing in a cloud infrastructure (Sec. III-A;
• T2D patients enrollment (Sec. III-B);
• Data indicators and features (Sec. III-C);
• Enrolled patient management (screening and follow-up): Self-Audit & Data Quality (Sec. III-D);
• Quality score for economic incentives (Sec. IV-D).

The framework is comprehensively evaluated on the a T2D dataset collected for this work. The details of the data collection and ground truth labeling are also discussed.

**FIGURE 2.**
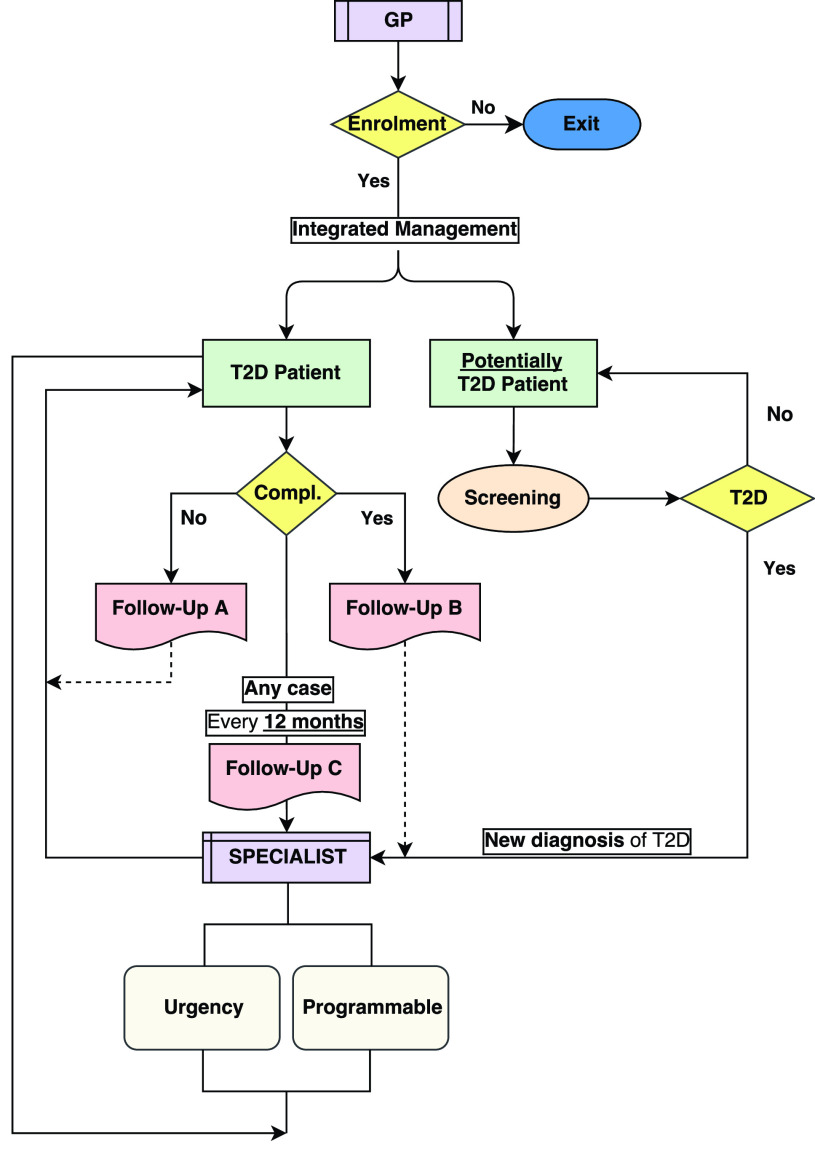
DSS for T2D patients integrated management care managing patient enrolment and treatment in the same conceptual flow and using the same data features.

GP membership to the system is free and there are no sanctions for GPs that do not intend to attend. GPs involved and patients cooperate to apply scientific guidelines. The flowchart in Fig. 2 shows a clinical DSS for T2D patients integrated management care.

### A. Data Collection of T2D Patients From EHRs and Data Sharing in a Cloud Infrastructure

The NMI platform manages a cloud computing project that, through the integration of GPs’ EHR databases, is able to realise: network medicine, audit process, data reporting, integrated management programs between GPs and specialists for treating chronic pathologies. NMI aggregates databases available from GPs in a unique standardized language and share them in a cloud platform. The database is available for transversal interoperability with other GPs, and for a vertical interoperability with several healthcare professionals of the NHS. The architectural features of the system meets stringent requirements: security and privacy, high reliability, ease of access and wide interoperability through the availability of flexible interfaces and standard communication protocols. Additionally, the system is equipped with services and tools which make it useful and usable by general practitioners, satisfying his/her needs for practice of the daily-life profession.

#### 1) Authentications and Authorizations

Data are security protected, through encryption during both transfer and storage. Data access is strictly allowed to only those that have the required permissions [43]. In particular, all connections and accesses are tracked and are subject to verification of the credentials and the possessed permits. A second level of access is regulated by a further 32-byte long key. The key is issued directly by Netmedica Italia to access different services according to the needs of the user.

#### 2) Interoperability Via Web Services Interface

The frameowrk allows a high degree of interoperability with other applications. It has an API interface to intercede directly with the cloud database. Most of the features of the Netmedica Cloud are provided by a Web Service Interface that exposes various functionalities through the publication of precise methods to be invoked. The name of the main interface is FIMMGwsdl. It is based on the Simple Object Access Protocol (SOAP) over HyperText Transfer Protocol (HTTP) and the default style is Remote Procedure Call (RPC).

The features offered by the web services can be grouped into the following macro categories:

• Access
• Writing
• Consultation
• Other services

Other services include features such as: notifications, patient report and delete record. These features and the relative methods are used by the extractor program that is specifically designed to receive the complete encrypted patient card. All patient cards are potentially analyzed, uploaded and stored, still encrypted, to the unified and normalized database.

#### 3) Data Extractor

The data centralization procedure of Netmedica Cloud is based on the specially developed automatic extractor software called NetDesk. All EHRs are first encrypted with the GP’s secret key and then transferred to the Netmedica cloud. The GP can install automatically the program with a wizard. Data are collected from the outpatient database, by applications that allow the extraction of clinical features of outpatients. The process of extraction normalizes the database according to a record layout defined in XML. After extracting and standardizing data into XML, through Web Services, data are forwarded to the cloud, where they are aggregated into a normalized database.

The extraction process takes place in 2 phases: first massive data extraction, successive extractions according to incremental logic. The GP can arrange the timing of the extraction (every 10, 30…minutes), even differing in a daily time when he is not using the PC. In clinics where more general practitioners work, it is possible to install the extractor on a network server that accesses the medical management software and the cloud database in multi-user mode. A GUI interface is available for managing user authorizations, scheduling the process, timing and extraction type.

#### 4) Database Platform

**FIGURE 3.**
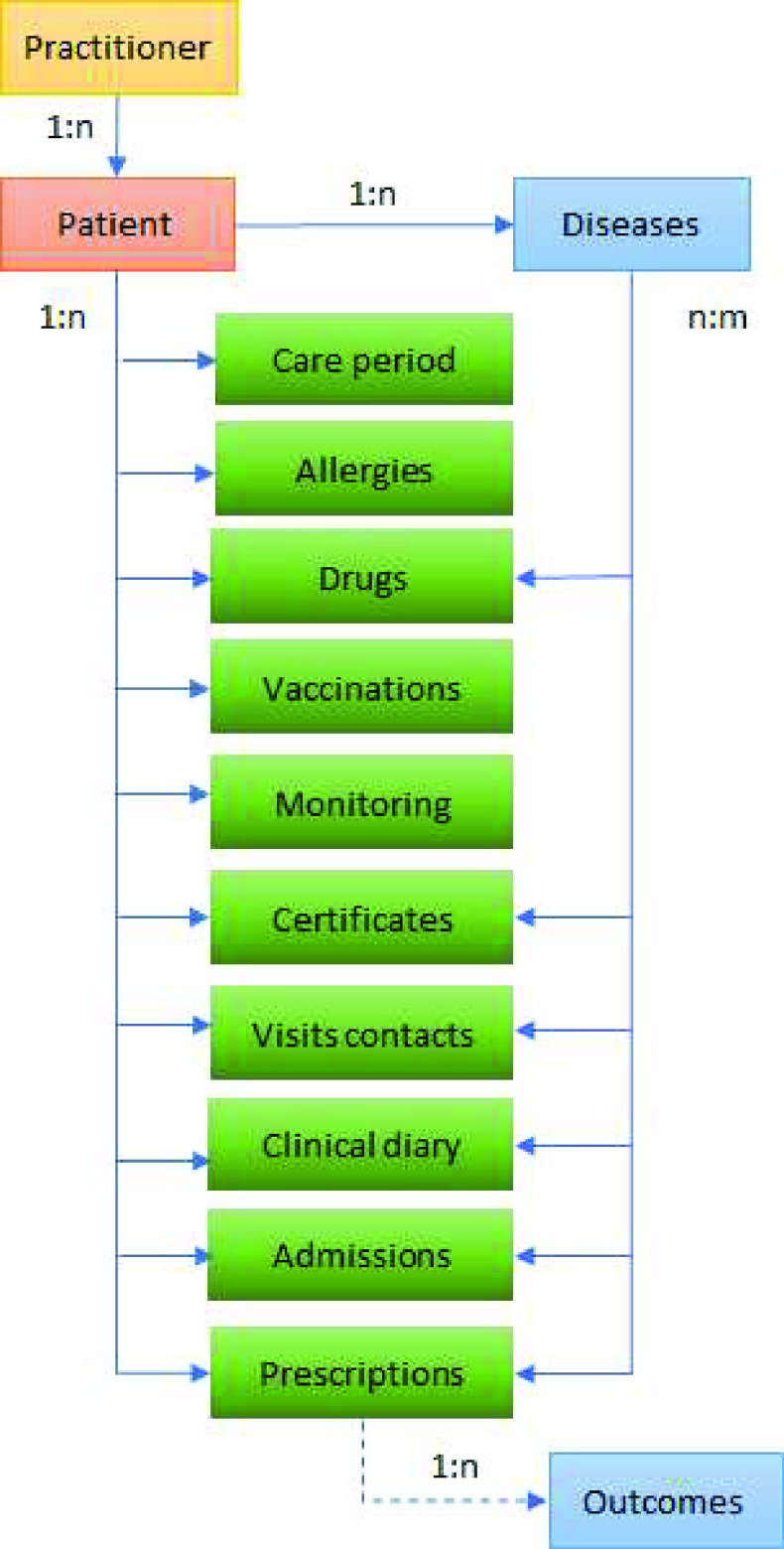
The available EHR fields in the database.

Through the WSDL, many services and applications communicate with the database. The database allows on-line sharing of care data, even among professionals that normally use different ambulatory management software. The organization of the data takes place in a patient centric manner and its structure has been designed as flat as possible. The figure 3 shows the available EHR field in the database.

A main patient registry table contains all the patient’s id informations. These informations are appropriately encrypted according to the key assigned to the physician.

### B. T2D Patients Enrollment

Once the GP is logged in with his/her own credentials, the system automatically proposes a list of T2D patients or potentially T2D (Table 1) extracted from the EHRs. Only T2D patients without major (uncontrolled) complications are automatically enrolled. Complications that lead to patient exclusions are:

• The presence of, at least, one of these pathologies: coronary heart disease (CHD), ictus, peripheral arterial disease, diabetic or hypertensive retinopathy;
• The presence of uncompensated diabetes: estimated in the presence of HbA1c > 8% in the last year;
• Insulin treatment in the last year.

**TABLE 1:** T2D Patients’ Enrolment DSS Output. Examples With Real (Anonymized) Data

					Update Indicators		Add Patient	
ID Patient	Type I	Type II	Exemptions	Drugs	Checks	Complications	Enrolment	Actions
A0001					*	*		Add
A0002					*	*		Remove
A0003		*					*	Remove
A0004		*	*		*		*	Remove
A0005			*				*	Remove
A0006					*			Add
A0007		*		*			*	Remove
A0008					*		*	Remove
A0009		*	*		*		*	Remove
A0010		*	*		*		*	Remove
A0011		*	*		*		*	Remove
…	…	…	…	…	…	…	…	…

Potential T2D patients may be automatically added by GPs. Potential T2D patients are subjects who, while not presenting the NHS code for T2D, have a high chance of developing T2D due to ongoing pharmacological treatment or specific clinical checks. With the “Add Patient” button, the physician starts collecting and maintaining informed all those patients who adhere to the integrated management path.

Table 1 shows an example of the output of the enrollment that suggests patients to be added or removed. The final decision is always performed by the GP.

### C. Data Indicators and Features

For a proper and correct implementation of a T2D integrated management system, it is necessary to define overall indicators for monitoring and evaluating treatment results. The care-quality indicators used to allow GPs to monitor and improve the care process of diabetic patients were taken from the international literature and the most important international guidelines on diabetes management. In order to evaluate the quality of the assistance provided and the conformity with the standards defined in the guidelines, it is crucial to identify process and outcome indicators to measure the achievement of the set goals. Specifically, the proposed indicators allows to control the activities of chronic care model and ultimately evaluate the capability of the integrated management pathway. Thus, these represent salient information to verify if and in which entity the totality of results has reached the set goals for improving the chronic care quality model.

The proposed care-quality indicators are:

• # 1.1 Indicator *“T2D Patients”* highlights the correspondence of the percentage of T2D patients with respect to total assisted patients enclosed in GP’s EHR. From here on, the following ratios will be calculated only respect to the total of enrolled T2D patients that have express the consent for adhering to integrated management system. The GP will schedule the visit of patients with T2D already under treatment and in according to the integrated follow up will update the EHR with indicator data required. The physician requires the determination of each indicator and records the value in the EHR.
• # 2.1 Indicator *“Diabetics with annual HbA1c”* is obtained from the ratio between T2D patients with HbA1c monitored minimum during last 12 months and the total number of T2D patients. This indicator expresses an adequate follow up of the patient.
• # 2.2 Indicator *“Diabetics with annual lipid profile”* is obtained from the ratio between T2D patients with lipid profile monitored minimum during last 12 months and the total number of T2D patients. It is demonstrated that LDL cholesterol reduction in diabetic patients reduces severe cardiovascular risks. The measurement cannot be calculated for triglyceride values >200 mg/dl.
• # 2.3 Indicator *“Diabetics with annual AP”* is obtained from the ratio between T2D patients with arterial pressure measured minimum during last 12 months and the total number of T2D patients. It is evidenced that the average prevalence of hypertension in diabetes is about 50%.
• # 2.4 Indicator *“Diabetics with BMI”* is obtained from the ratio between T2D patients with Body Mass Index measured and the total number of T2D patients. This indicator is indispensable to evaluate the effectiveness of therapy and can suggest a cardiovascular risk factor. The physician processes BMI and records the values in the EHR and, finally, performs an educational reinforcement.
• # 2.5 Indicator *“Diabetics with Waist”* is obtained from the ratio between T2D patients with Waist measured and the total number of T2D patients. This indicator is indispensable to evaluate the effectiveness of therapy and can suggest a cardiovascular risk factor. The physician processes this value and records it in the EHR and, finally, performs an educational reinforcement.
• # 2.6 Indicator *“Diabetics with annual Microalbuminuria”* is obtained from the ratio between T2D patients with microalbuminuria measured minimum during last 12 months and the total number of T2D patients. Microalbuminuria is an early marker of diabetic nephropathy when there is still hope for reversibility or arresting progression.
• # 2.7 Indicator *“Diabetics with annual Creatinine”* is obtained from the ratio between T2D patients with creatinine measured minimum during last 12 months and the total number of T2D patients. This indicator is a very sensitive and specific index of glomerular insufficiency. It is important not only to diagnose kidney failure, but also for any contraindications to the use of nephrotoxic drugs.
• # 3.1 Indicator *“Diabetics with HbA1c* ≤ 6.5 *%”* is obtained from the ratio between the number of T2D patients with latest registered value of HbA1c ≤ 6.5% and the total number of T2D patients. Values below 6.5% prevent the onset of complications.
• # 3.2 Indicator *“Diabetics with LDL* ≤ 130 *mg/dl”* is obtained from the ratio between the number of T2D patients with latest registered value of LDL cholesterol ≤ 130 mg/dl and the total number of T2D patients. Reduction in LDL cholesterol values reduces cardiovascular risk. Physician reinforces life fitness education, evaluates therapeutic strategy after stratification of cardiovascular risk.
• # 3.3 Indicator *“Hyp diabetics with AP* ≤ 130 */80 mmHg”* is obtained from the ratio between T2D and hypertensive patients with AP registered value ≤ 130/80 mmHg and the total number of T2D patients. Antihypertensive therapy in diabetic subjects, if effectively conducted, reduces micro and macrovascular complications. The GP monitors the values of the Arterial Pressure and, eventually, modifies the therapy.

Table 2 shows aggregated data collection under the evaluation period. For each indicator, the (*“Ratio”*) achieved by the GP is shown. The *“Ratio”* estimates the correlation between the single indicator and the entire patient population.

**TABLE 2:** Care Quality Indicators Under Evaluation for Improving the Clinical Performance. Data From a Single GP

#	Description	Num	Den	Ratio	Target	Distance	Mean	LAP
1.1	T2D Patients	74	1501	4.93%	3%	64.33%	6.17 %	150
2.1	Diabetics with annual HbA1c	43	74	58.11%	70%	−16.99%	66.41 %	100
2.2	Diabetics with annual lipid profile	56	74	75.68%	60%	26.13%	46.64 %	100
2.3	Diabetics with annual AP	24	74	32.43%	90%	−63.96%	63.35 %	100
2.4	Diabetics with annual BMI	5	74	6.76%	70%	−90.35%	72.73 %	50
2.5	Diabetics with annual waist	0	74	0	50%	−100%	53.95 %	50
2.6	Diabetics with annual microalbuminuria	10	74	13.51%	50%	−72.97%	31.00 %	100
2.7	Diabetics with annual creatinine	59	74	79.73%	60%	32.88%	41.25 %	50
3.1	Diabetics with HbA1c ≤ 6.5%	35	74	47.3%	25%	89.19%	51.95 %	100
3.2	Diabetics with LDL ≤ 130 mg/dl	43	74	58.11%	20%	190.54%	37.44 %	100
3.3	Hyp diabetics with AP ≤ 130/80 mmHg	5	50	10%	20%	−50%	37.68 %	100

The percentage Ratio achieved by the physician for each indicator is compared with the expected *“Target”* established by the diabetes project, and its positive or negative *“Distance”* from the target is calculated. At this step, in order to assign the overall *“Acceptable Level of Performance (LAP)”* score, only if the target is exceeded by the ratio, the LAP score provided by each indicator is assigned. The *“Mean”* value of the indicator of all GPs participating in the project is also reported.

Every GP can also consult the following tables that report for information purposes the use of antidiabetic drugs (Table 3) and the detection of complications(Table 4). For each indicator, the GP can compare his performance with correspondent average value reached by all GPs participating in the project. In particular, the information reported in Tables 3 and 4 refers to only one GP. However, the “Mean” value represents the average of all GPs participating in the project. Although these Tables represent standard medical information related to antidiabetic drugs subministration and complications in act, it allows the comparison between the incidence of each indicator for each GP and the average value reached by all GPs participating in the project. Data indicators were used specifically to determine the economic incentives, but globally they may help every single GP to improve diabetes care by focusing on specific complications and drugs that have a greater incidence for them.

**TABLE 3:** Antidiabetics Drugs Subministration. Data From a Single GP

Description	Num	Den	Ratio	Mean
Diet treatment	49	75	65.33%	20.67%
Insuline treatment	2	75	2.67%	19.46%
Metformin treatment	21	75	28%	44.71%
Sulfaminide treatment	6	75	8%	13.44%
Acarbose treatment	1	75	1.33%	5.03%
Pre cost treatment	3	75	4%	24.01%

**TABLE 4:** Complications in Act. Data From a Single GP

Description	Num	Den	Ratio	Mean
Ischemic cardiopaty	3	75	4%	17.27%
AMI	1	75	1.33%	2.84%
Revascularisation	1	75	1.33%	1.82%
Claudicatio	2	75	2.67%	0.37%
TIA	1	75	1.33%	1.74%

These indicators, together with a subset of the EHRs, are used to perform a machine-learning-based evaluation of chronic care quality (Subsection IV-B).

### D. Enrolled Patient Management (Screening and Follow-Up)

The patient management comprises the following steps.

#### 1) Potentially Diabetes Patient Screening (Figure 2)

To enroll patients that are suspected to develop T2D, GPs inspect and record lifestyle habits (eating habits, alcohol, smoking, physical activity, work activity), measure and record weight, height, BMI, AP, waist and calculate and record the cardiovascular risk score [44]). The screening of potentially diabetic patients was performed periodically by evaluating the fasting plasma glucose test (more cost-effective than HbA1c and OGTT) in subjects over 45 years. GPs perform a fasting plasma glucose test to discriminate diabetes in subjects with BMI > 25 kg/m^2^ and at least one or more of the following conditions in subjects under 45 years:

• Physical inactivity;
• 1st degree familiarity with T2D;
• Belonging to a high-risk ethnic group;
• Arterial hypertension (≥ 140/90 mmHg) or antihypertensive therapy in act;
• HDL cholesterol < 35 mg/dl and/or triglycerides >250 mg/dl;
• Past diagnosis of gestational diabetes or infant birth with > 4 kg weight;
• Previous diagnosis of Impaired Glucose Tolerance (IGT) or Impaired Fasting Glucose (IFG), HbA1c 42–48 mmol/mol;
• Insulin resistance;
• Clinical evidence of cardiovascular disease (AMI, stroke, claudicatio, etc.) according to a cardiovascular risk score [44].

In the absence of the previous criterion, screening should start at the age of 45 years. If the blood glucose is not diagnostic for diabetes (< 126 mg/dl), screening should be repeated at least three years, considering a more frequent test for subjects with dysglycemia (>100 and < 126 mg/dl). In addition to diabetes, other dysglycemia patterns are known. To define these conditions, however, the use of the term “pre-diabetes” may be misleading and thus not recommended. Hence, the following values of the main glycemic parameters should be considered, as they identify subjects at risk of diabetes and cardiovascular disease [45]–[47]:

• Fasting blood glucose 100-125 mg/dl (IFG)
• 2-hour glucose after OGTT 140-199 mg/dl (IGT)
• HbA1c 42–48 mmol/mol (only with IFCC aligned assay)

#### 2) New Diagnosis of T2D (Figure 2)

GP makes a general visit and prescribes the first indications on lifestyle (diet, physical activity, smoking abolition, etc.). Moreover, GP considers the opportunity to initiate drug therapy (metformin, if not contraindicated) and to send the patient to the dietician. Finally, GP requires investigations for the first diagnostic check-up by the specialist:

• HbA1c, total cholesterol, HDL, LDL, triglycerides; creatinine, AST, ALT, GGT, blood count;
• Microalbuminuria;
• Full urine examination;
• ECG (and cardiologic examination at discretion);
• Fundus oculi.

Then GP sends the patient to diabetes center to perform:

• Diagnostic overview;
• Specialists clinical staging and any complications;
• Certification for diabetes exemption;
• Compilation, if necessary, of the therapeutic plan, assessment of care criticality, individual or group therapeutic education planning.

Finally, depending on the clinical condition, the specialist:

• Starts not complicated T2D patients’ follow-up (follow-up A);
• In agreement with GP, approves the care plan for insulin-dependent diabetes and/or complications and/or inadequate control (follow-up B).

#### 3) Follow-Up a of T2D Patient Without Complications (Figure 2)

The care quality is also based on specific follow up for every enrolled patients. The proposed NMI system requests every GP to register data relevant to the follow up process, which are automatically retrieved when requested by the GP. GP conducts a general medical examination: history to detect urinary, visual, cardiovascular and neurological disorders (erectile dysfunction, muscle cramps, paraesthesia, skin disorders, etc.); peripheral wrists, vascular soffits, heart rate, tendon reflexes, tactile sensitivity examination, skin and feet examination.

Every 3 months within GP’s dedicated outpatient clinic:

• Body weight, BMI and waist;
• AP;
• Evaluation of the blood glucose control performed by the patient.

GP each year prescribes: HbA1c, blood glucose and any other examinations based on clinical judgment and/or how agreed with the diabetic specialist, full urine examination, microalbuminuria, clearance, creatinine, total cholesterol, HDL, triglycerides, ECG.

GP every 2 years prescribes Fundus oculi and record results in the EHR.

#### 4) Follow-Up B of T2D Patient With Stabilized Complications (Figure 2)

Every 6 months GP sends patients with stabilized complications to the diabetes center:

• Activities suggested by Follow-up A;
• In relation to clinical needs, diabetic pathology specialist (including examination aimed at finding lesions of the feet).

Depending on the intervals programmed for insulin-treated diabetics and/or with evolving complications and/or inadequate control, GP sends the patient to the diabetes center in case of:

• Periodic inspection, if provided by the individual care path, agreed with the diabetic team;
• Social-welfare criticisms that lead to erroneous or non-therapeutic adherence;
• Failure to maintain agreed therapeutic goals, especially if present:
  • Severe and/or repeated hypoglycemia;
  • Rapidly evolving neurological, renal, ocular or macrovascular complications;
  • Diabetic foot (ulceration or infection);
  • Pregnancy in diabetes, gestational diabetes.

Moreover, diabetic center can:

• Perform further specialist examinations (ecocolordoppler, angiographic exams, percutaneous oximetry, electromyography, retinography, etc.);
• Activate additional therapeutic treatments;
• Agree with GP for any personalized clinical-therapeutic-assistance plan (in the case of diabetes with evolving complications);
• Manage with a multidisciplinary approach, and according to organizational resources, patients who have:
  • Severe metabolic instability;
  • Neurological, renal, ocular or macrovascular complications that are rapidly evolving;
  • Diabetic foot (ulceration or infection);
  • Erectile dysfunction;
  • Pregnancy in diabetes, gestational diabetes.

#### 5) Follow-Up C of all Patients With T2D (Figure 2)

Every 12 months, GP sends patient to the diabetic centre to allow annual screening, sharing all the available data. If the clinical conditions are stable, the annual renewal of the therapeutic plan will be reported directly by GP, otherwise a new one will be planned.

The integrated management provides a specialist’s visit in the following cases (beyond the new diagnosis and annual screening):

• **Urgency:**
  • Acute metabolic deficit;
  • Repeated episodes of hypoglycaemia;
  • Pregnancy in diabetic women;
  • Appearance of foot ulcer or ischemic and/or infectious lesions at the lower extremities.
• **Programmable:**
  • Repeated glycemic fasting > 180 mg/dl;
  • HbA1c > 6.5% in two consecutive determinations;
  • Appearance of clinical signs related to complications.

The NMI infrastructure makes data processing possible for individual physicians or diabetic team for every one of the previously described follow up. Data processing is focused on audit tools for improving the use of the medical tool by the physician and on specific local projects aimed at the treatment of chronic diseases or prescriptive appropriateness. The processing system is built in such a way as to maintain historical memory of past elaborations for reporting data or for checking trends. Using a standard data format, regardless of the record software used by the physician, facilitates the collection, processing, and sharing of information.

### E. Self-Audit & Data Quality

Through self-audit, the physician can evaluate his performance compared to colleagues on a set of standard indicators that can be subdivided into four areas:

i. *Recording completeness:* The accuracy of the collected data, the presence of the main outpatient data (AP, BMI, etc.) and the recording of laboratory results in numerical format are evaluated;
ii. *Adherence to prevalence:* Distances from the prevalence of the major chronic conditions are shown to the physician. Patients potentially affected by the pathologies under investigation are reported by examining therapeutic prescriptions, examinations carried out, exemptions granted etc.;
iii. *Treatment of chronic diseases:* The physician is evaluated on the main indicators of fitness identified by the international guidelines as compared to the main chronic diseases;
iv. *Contact intensity:* In addition to the quality of the recordings, the amount of these records is also measured. The purpose is to document the activities of the physician.

Table 5 shows the indicators under LAP score evaluation for each patient. For the indicators: HbA1c, LDL cholesterol and Pressure (only if the patient is hypertensive), the cell assumes green or red colour, as the result for the examination falls within the thresholds established by project. For indicators: BMI, Waist, Microalbuminuria and Creatinine, the star symbol in green is displayed if the data is recorded with a coherent numeric value within the period indicated by the project.

**TABLE 5:** Self Audit & Data Quality Aggregated Visualization. Sample Data for a GP. Red Show Warning Data for a Particular Feature of an Enrolled Patient

Patient	HbA1c	LDL	Press.	BMI	Waist	Micr.	Creat.
Patient 1	6.54	150					$\bigstar $
Patient 2	8.46	120	80/150				$\bigstar $
Patient 3	7.27	117		$\bigstar $			$\bigstar $
Patient 4		123					$\bigstar $
Patient 5	6.82	144				$\bigstar $	$\bigstar $
$\ldots $	$\ldots $	$\ldots $	$\ldots $	$\ldots $	$\ldots $	$\ldots $	$\ldots $

The system finally has the complete set of features for every T2D patient (Table 6) where all the indicators required by the project are displayed.

**TABLE 6:** T2D Patient’s Feature Set. Example From a Real Anonymous Patient

PATIENT DETAILS		
Glycated hemoglobin	Not registered	
Total cholesterol	Not registered	
HDL cholesterol	Not registered	
Triglycerides	Not registered	
LDL cholesterol	Not registered	
Blood Pressure	21/01/2016	80/130
Weight	21/01/2016	70
Height	21/01/2016	156
BMI	21/01/2016	28.76
Waist	21/01/2016	98
Microalbuminuria	Not registered	
Creatinine	Not registered	
Exemptions:		
ND	E00	
ND	013_R	
ND	E10	
Pharmacological treatment:		
Glucophage * 30 cps 500 mg	14/01/2016	Metmorfin
Lifestyles:		
Smoking habit	Not detected	
Cigarettes day		
Alcohol consumption		
Alcohol type		
Physical activity		
Physical activity type		
Macrovascular Complications:		
Ischemic Cardiopathy	Not Affection	
AMI	Not Affection	
Revascularization	Not Affection	
Claudication	Not Affection	
TIA	Not Affection	
Stroke	Not Affection	
Angina	Not Affection	
Eye Examination:		
Eye examination	29/02/2012	Performed
Retinopathy	Not Affection	
Blindness	Not Affection	
Examination of the Foot:		
Ulcers	Not Affection	
Amputations	Do not suffer	
Renal complications:		
Nephropathy	Not Affection	
Dialysis	Not subjected	
Educational Reinforcement:		
Power	Not done	
Motor activity	Not done	
Self control	Not done	
Foot Prevention	Not done	
Verified Glycemic Control	Not done	
Findings:		
Foot Inspection	Not done	
Uricemia	Unregistered	
AST	Not registered	
GGT	Not registered	
Blood count Formula	Not done	
Cardiovascular risk	Not registered	
Specializations:		
ECG	Not done	
Visit diabetes	Not done	
Cardiological visit	Not done	
Eye examination	Not done	
Eyeglass Fund	Not done	
Nephrologic visit	Not done	
Neurological visit	Not done	
Date enrolled:	16/09/2015	

If a data used in the dataset for the care quality and economic incentives evaluation is not registered, the relative row is shown in grey. Process indicators are continuously monitored. If a data is not collected, the physician is alerted by the system to understand the nature of the problem. If a data is collected incompletely, a different type of notification is sent proactively to the physician.

## IV. DSS Analysis on a Clinical Use Case for Quality Care Evaluation

In this section, the results of care quality evaluation relevant to 2018-2019 are presented. The quality of care was evaluated for every GPs based on T2D Patient’s feature set (see Table 6), with the main purpose to foster care-delivery quality improvement. The dataset comprised of a total of 41237 patients. The dataset annotation was firstly described in Section IV-A, while Section IV-B described the Machine Learning approach and results. Finally, Section IV-D reported the impact of the proposed DSS in terms of economic incentives.

### A. Dataset Annotation

A subset of the dataset comprised of 1780 patients was extracted from the entire dataset (41237 patients) and was manually annotated by experts. This distribution was equally balanced across the five follow-up phases (19% Potentially diabetes patient screening, 21% New diagnosis of T2D, 20% Follow-up A of T2D patient without complications, 22% Follow-up B of T2D patient with stabilized complications, 18% Follow-up C of all patients with T2D). Each of the follow-up phase (i.e. Potentially diabetes patients screening, a new diagnosis of T2D, Follow-up A of T2D patient without complications, Follow-up B of T2D patients with stabilized complications and Follow-up C of all patients with T2D) described in Section III-D was manually annotated by a team of 10 experts (5 from GPs leading group and 5 from diabetes centers). The experts evaluated the chronic care quality according to a 5-Likert ordinal scale [48] ranging from level 1 (Excellent) to level 5 (Poor). The labels may be affected by the inter-observer/expert variability: the experts can evaluate the chronic care quality in a different way based on their different motivation, experience and background knowledge. For this reason we have alleviated this problem by averaging the response of the ten expert GPs according to a majority vote approach. As future work, we are considering to rank the label according to a confidence level [49] and to further minimize the inter-rater variability by developing a Multi-task learning approach and maximizing a consensus among annotators.

The input of the classifier was represented by the T2D patient’s feature set described in Table 6. The majority vote of the expert ratings represented the chronic care quality ground-truth. The final dataset was comprised of a total of 1780 observations balanced across the five follow-up phases. Two years interval (2018-2019) was considered for learning and evaluating the ML model while the data of the following 6 months were used to evaluate the improvement of the economic incentives.

### B. Machine Learning Approach

The Random Forest (RF) [50] was employed for classifying chronic care quality. RF is a variant of bagging proposed by [50] and consists of an ensemble of decision trees (DT) (i.e., $n^{\circ }$ of DT) generated by an independent identically distributed random vectors. RF is developed by sampling from the samples, from the features (i.e., $n^{\circ }$ of features to be selected) and by changing two tree-parameters (i.e., max $n^{\circ }$ of splits and max $n^{\circ }$ of size) [51]. The splitting features for each node was computed according to the Gini index metric.

The model was built using Azure Machine Learning Studio and was deployed as web services on the proposed Service-oriented architecture. The 10 cross-validation (CV) procedure was implemented, dividing all datasets into 10 folds and selecting iteratively nine folds for training and one fold for testing. This procedure was stratified across the five follow-up phase. The optimization of the RF hyperparameters (i.e., $n^{\circ }$ of RT, max $n^{\circ }$ of splits, $n^{\circ }$ of features to select at random for each decision split) was performed implementing a grid-search and optimizing the macro-recall in a nested Fivefold Cross-Validation.

### C. Machine Learning Results

The RF achieved an overall accuracy (averaged over the 10 fold) of 98%±2% and macro-recall of 96%±1%. This result suggests how there is a close dependency between the indicators displayed in Table 6 and the chronic care quality ground-truth. Moreover, these indicators are informative for each of the follow-up phases of T2D patients. Accordingly, the proposed DSS might be exploited to support all GPs over time by providing incentives for moving from one class to another (i.e. from Poor to Excellent) with the main objective of improving the chronic care quality.

Furthermore, the extracted results pointed out how the proposed DSS, along with the economic incentives, brought to a significant improvement of class A (i.e. an increasing number of patients in class A [Follow-up A of T2D patient without complications]), with more than 12% of the increase in the first 6 months. This result refers to a six-month prospective outcome of the selected samples (1780 patients). The total incentive costs are not comparable with the impact of the care quality on quality life and reduction of TDM consequences over time and their social costs.

### D. Impact of the Proposed DSS: Quality Care Evaluation for Economic Incentives

By selecting the LAP column, GP observes the LAP schedule and the incentives calculated for the reference period (Table 7). By selecting the Diabetics column, the enrolled patients’ schedule is shown. The GP visualizes the overall indicators that determine the LAP score. Moreover, by selecting the patient’s name, GP access to every detailed indicator.

**TABLE 7:** Data Delivered Consultation

Date	Diabetics	LAP	Enrollement inc.	LAP inc.	Total inc.
2019	72	500	3. 600,00 €	2.160,00 €	5.760,00 €

GPs receive the remuneration of 50 € per patient enrolled. In addition standard remuneration, the GP receives annually a bonus related to the LAP score achieved. For determining the overall “LAP Score”, GP’s performance is compared with the project “Target” for each indicator. If the target is reached or exceeded, the “LAP” for the indicator is assigned (see Table 2).

Thus, the LAP score determines a further economic remuneration (LAP inc.) that can be 30, 40 or 50 € per patient/year, as showed in Table 8.

**TABLE 8:** LAP Score Incentives

LAP	LAP inc. per patient
From 300 to 599	30 €
From 600 to 5799	40 €
From 800 to 1000	50 €

The bonus is accumulated over the test period and can be used in similar cases both from the economic point of view or as a performance indicator that can be transformed into different loyalty programs.

## V. Limitations and Future Work

The proposed DSS lie the foundation for enhancing the sharing of information among other GPs by allowing a more planning clinical diagnosis and analysis and continuity of assistance to patients who need it. The experimental results show how the ML model is able to support the GP while accurately predicting the chronic care quality based on specific indicators selected by GPs. However one important limitation of the proposed pilot study may be the specific focus on diabetes care quality. In medicine, all the guidelines consider the management of the pathology in standard conditions or with the most frequent and known comorbidities and variables; then it is up to the physician’s preparation and awareness to adapt the recommendations of the guidelines to the specificity of the individual patient, also taking into account the infinite variability of individual clinical conditions. Future work may be addressed to (i) validate the proposed DSS for the management of different chronic diseases and (ii) generalize and standardize these quality indicators for the prediction of chronic care quality related to different pathology. Accordingly, another interesting future direction may be addressed to store GP’s feedback temporally. In this scenario, a sequential ML model can be exploited to temporally model the ground-truth chronic care quality observations.

## VI. Conclusion

The paper presented and tested on a real clinical use-case scenario and over time a comprehensive framework for supporting the GPs during the diabetes early detection & enrollment stage. In particular, the work proposed an integrated chronic care model based on machine learning and data sharing between GPs and diabetes centers, as the main core of a decision support system. The quality care evaluation in a clinical use-case scenario demonstrated how the empowerment of the GPs through the use of the platform (integrating the proposed DSS), along with the economic incentives, may speed up the improvement of care. The National Sanitary System and its regional agencies are funding the GP’s incentives. The overall investment is based on the concept that prevention is less expensive than intervention. One of the goal of the chronic care models described in this paper is to prove a long-term positive balance on the overall care strategy and budget, both in economic value and in quality of life.
